# Comparison of multimodal attract-and-kill formulations for managing *Drosophila suzukii*: Behavioral and lethal effects

**DOI:** 10.1371/journal.pone.0293587

**Published:** 2023-12-07

**Authors:** Arun Babu, Elena M. Rhodes, Cesar Rodriguez-Saona, Oscar E. Liburd, Conor G. Fair, Ashfaq A. Sial

**Affiliations:** 1 Department of Entomology, University of Georgia, Athens, Georgia, United States of America; 2 Entomology and Nematology Department, University of Florida, Gainesville, Florida, United States of America; 3 Department of Entomology, Rutgers University, New Brunswick, New Jersey, United States of America; University of Carthage, TUNISIA

## Abstract

Attract-and-kill (A&K) is a potential alternative control tactic for managing the invasive spotted-wing drosophila, *Drosophila suzukii* Matsumura. Here, we compared the efficacy of two novel A&K formulations based on proprietary blends–ACTTRA SWD OR1 (henceforth OR1) and ACTTRA SWD TD (henceforth TD)–in managing *D*. *suzukii*. Using two-choice bioassays, we compared OR1 and TD for their relative attractiveness to adult *D*. *suzukii*. Additionally, we tested how the addition of (1) a red dye (visual cue) and (2) the insecticide spinosad (Entrust™) to the OR1 and TD formulations influenced the attraction of adult *D*. *suzukii* in the presence of blueberry fruits. Finally, complementary laboratory efficacy (no-choice) bioassays were conducted to assess the mortality of adult *D*. *suzukii* exposed to OR1 and TD. A direct comparison between TD and OR1 formulations indicated the TD formulation was ~8 times more attractive than OR1. Adding a red dye to the TD or OR1 formulation did not significantly alter the attraction or mortality of adult *D*. *suzukii* compared to the formulation without a dye. Similarly, irrespective of dye status, adding spinosad to either the TD or OR1 formulation did not alter the adult *D*. *suzukii* behavioral response to these formulations but resulted in significantly higher *D*. *suzukii* mortality. Overall, the TD formulations resulted in significantly higher, or at least comparable, mortality to the OR1 formulations. In summary, our laboratory results demonstrated the higher efficacy of a TD-based A&K product in managing *D*. *suzukii* over its well-tested predecessor, the OR1 formulation, confirming its potential as a new behavioral tactic against this pest.

## Introduction

Insecticide-based tactics are the primary method for managing many insect pests of agricultural crops, and multiple alternate management methods that fit the integrated pest management (IPM) model have been developed to reduce the reliance on insecticides [1, 2]. These IPM-based management methods can be crop and pest-specific and often vary in their effectiveness and scope [3]. The attract-and-kill (A&K) tactic is one of the potential alternate management strategies that can substantially reduce insecticide application volume. This technique involves the use of a combination of an attractive semiochemical blend and a lethal insecticide [4]. The semiochemical blend attracts the target insects to the point source. Upon interacting with the formulation, the insects acquire the insecticide through contact or ingestion, leading to their mortality [5]. The A&K formulation is applied as discrete point sources across the fields, compared to a conventional insecticide cover spray application; therefore the volume of insecticide applied per unit area is significantly reduced when deploying the A&K formulation. This characteristic of A&K tactics limits the non-target impact of the insecticide and insecticide residue problems in harvested crops [6].

The invasive spotted-wing drosophila, *Drosophila suzukii* Matsumura (Diptera: Drosophilidae), is a major insect pest with great economic significance for small fruit growers worldwide [7–10]. Depending on *D*. *suzukii* population pressure and environmental factors, frequent, often weekly or biweekly insecticide applications are the primary management tactic for this pest [11, 12]. However, alternate management methods, including A&K tactics, have been developed or are being investigated [2, and citations therein]. One promising experimental A&K tactic for managing *D*. *suzukii* is ACTTRA SWD (ISCA Technologies Inc., Riverside, CA). ACTTRA SWD formulations include a group of experimental formulations based on a proprietary slow-release SPLAT (Specialized Pheromone and Lure Application Technology) matrix [6]. These multimodal formulations consist of a blend of olfactory attractants, a red dye for adult *D*. *suzukii* visual attraction, and sugars as phagostimulants. A SPLAT-based A&K formulation similar to ACTTRA SWD named HOOK SWD has previously been tested for efficacy in managing *D*. *suzukii* in small fruit crops [13–16]. HOOK SWD contains ACTTRA SWD OR1 (henceforth OR1) as an attractive blend and technical-grade insecticide spinosad as the killing agent. In comparison to HOOK SWD, the OR1 formulation is considered an adjuvant because it contains the same attractive blend but without the insecticide; thus, OR1 can be mixed with any insecticide before application. Recent studies suggested that the application of HOOK SWD in blueberry crops resulted in a 2‒8 times reduction of fruit infestation by *D*. *suzukii* compared to the control plots [14]. Similarly, a 2‒5 times reduction in fruit infestation was observed in a commercial raspberry field when HOOK SWD was applied weekly or biweekly after a standard insecticide cover spray [14]. These results suggest the potential of HOOK SWD, i.e., an OR1-based A&K formulation, as a behavior-based strategy for *D*. *suzuki*i management in small fruit crops. However, the field efficacy of HOOK SWD can be variable in time and space [16]; thus, new, more effective A&K formulations need to be identified.

Compared to HOOK SWD and its attractive blend OR1, the ACTTRA SWD TD (henceforth TD) is a newer adjuvant formulation (ISCA Technologies, Inc.). While the OR1 formulation contains a blend of five attractants, the TD blend consists of nine different attractants [A. Mafra-Neto, personal communication]. A laboratory study conducted to identify the relative attraction of adult *D*. *suzukii* to the OR1 and TD formulations against common *D*. *suzukii* host fruits indicated that the TD formulation exhibited a higher potential than OR1 to compete with fruit odors for attracting adult *D*. *suzukii* [17]. Although these results predict a better potential of TD than OR1 in managing *D*. *suzukii*, a direct comparison between OR1 and TD formulations in attracting adult *D*. *suzukii* is unavailable to confirm the above prediction. Thus, new information is needed to assess the performance of the TD formulation over the OR1 formulation. Similarly, although information on the efficacy of TD and OR1 formulations mixed with various insecticides on adult *D*. *suzukii* mortality is available [18, 19], in these studies, OR1 and TD formulations were tested in separate experiments. Thus, an experiment comparing the efficacy of these formulations side-by-side is essential to assess the potential of one formulation in comparison with the other in managing *D*. *suzukii*.

Along with the volatile cues emanating from the host, *D*. *suzukii* adults are also known to utilize visual cues for host finding [20]. Accordingly, together with the characteristics of volatile blends, color may also be an important factor in deciding the efficacy of ACTTRA SWD-based A&K formulations. For instance, several studies aimed to optimize trap designs for improving adult *D*. *suzukii* capture suggest that red or black colors had the highest captures [21, 22]. In addition, a preference for yellow and purple colors has also been observed [23, 24]. Conforming to the above findings, the ACTTRA SWD formulations, by default, contain a red dye for insect visual attraction. However, recent studies on the visual perception of adult *D*. *suzukii* suggested that rather than the color appearance, adults rely on the color contrast between the fruits and their background for detecting the host fruits [20] and that adult *D*. *suzukii* have a limited ability to distinguish red color [25]. Thus, further research is needed to assess the advantage of adding a red dye to enhance adult *D*. *suzukii* visual attraction to the ACTTRA SWD formulation and in the resulting adult mortality.

A commercial insecticide formulation added to an attractive blend can compromise the resulting A&K formulation’s ability to attract the target insect pest. Either the insecticide active ingredient(s) or other components in the commercial formulation can interact with the attractants, resulting in an overall change in the formulation’s ability to attract the target insect, or it can interfere with the subsequent insect landing and contact with the formulation containing the killing agent [5]. The organic insecticide spinosad (Entrust SC, Dow AgroSciences LLC, Indianapolis, IN) is the default choice as a killing component for mixing with ACTTRA SWD to prepare an A&K formulation [26]. Moreover, HOOK SWD also contains spinosad as the killing agent [13–15]. However, to confirm the suitability of spinosad as the killing agent with the ACTTRA SWD formulations, the behavioral response of adult *D*. *suzukii* to ACTTRA SWD formulations when added with spinosad compared to that of a corresponding blank formulation needed testing.

Our goal was to compare two ACTTRA SWD formulations—the OR1, an older formulation, with the TD, a newer and more complex formulation in terms of the number of attractants—for managing adult *D*. *suzukii*. We tested adult *D*. *suzukii* attraction to these formulations with and without an added insecticide against each other, and a water control by using two-choice bioassays. Additionally, using two-choice assays, we tested how the addition of (1) a dye and (2) an insecticide (spinosad) to both the OR1 and TD formulations influence the attraction of adult *D*. *suzukii* to these formulations in the presence of blueberry fruits. Finally, complementary laboratory-based efficacy (no-choice) bioassay tests were conducted to assess the mortality of adult *D*. *suzukii* exposed to these treatments. For broad comparison, HOOK SWD and spinosad cover spray treatments were added to the treatment lists of the above efficacy trials.

## Materials and methods

### Insect sources

The adult *D*. *suzukii* individuals used in our two-choice and efficacy bioassays were reared from the stock colonies maintained at participating laboratories located in the following three US states: Georgia (GA), New Jersey (NJ), and Florida (FL). For GA studies, flies selected were reared from a laboratory colony maintained at the University of Georgia, Athens, GA. This colony originated from a *D*. *suzukii* population collected from blueberry fields of Clarke Co., GA, in the summer of 2013. Similarly, for the bioassays conducted at NJ, adult flies from a colony maintained at the Rutgers P.E. Marucci Center (Chatsworth, NJ) were used. This colony was also initiated in 2013 by using flies that eclosed from *D*. *suzukii*-infested blueberries collected from grower fields around Atlantic Co., NJ. For FL studies, flies used in the experiments were obtained from a laboratory colony housed at the University of Florida Small Fruit and Vegetable IPM Laboratory (Gainesville, FL). The colony was initially set up in late spring 2016 from *D*. *suzukii*-infested blueberries from plantings in Alachua and Bradford counties. All colonies were periodically mixed with *D*. *suzukii* populations from the wild to maintain the colony’s health.

Flies were reared on the standard fly diet [27] in sterilized 117-mL square-bottom polypropylene bottles (Genesee Scientific, San Diego, CA). Each bottle was provided with ~37 mL of diet, added with a pinch of active baker’s yeast, and capped with cellulose acetate plugs. The colonies were maintained in growth chambers (Percival Scientific, Perry, IA, USA), held at 24 ± 2°C, 60% RH, and 14:10 L:D.

### ACTTRA SWD formulations

Here, we compared the attractiveness and efficacy of two ACTTRA SWD formulations, namely, ACTTRA SWD OR1 (OR1) and ACTTRA SWD TD (TD), for managing adult *D*. *suzukki*. Both TD and OR1 formulations are intended to be marketed as adjuvants and lack an insecticide component. Thus, before deploying it as an A&K strategy, growers must mix these adjuvants with an appropriate insecticide. On the contrary, HOOK SWD is an experimental A&K formulation consisting of a blend of attractants (same as OR1 formulation) and a killing agent already mixed into the formulation. In all three A&K formulations, the killing agent was spinosad (Entrust™ 22.5 SC, Dow AgroSciences LL) incorporated at 0.25% (v/v). All the formulations (OR1, manufacturing date: 14 May 2021, batch ID: 2458857; TD, manufacturing date: 27 May 2021, batch ID: 2459361 Blend B091; and HOOK SWD, manufacturing date: 12 March 2021, batch ID: C097) were kindly provided by ISCA Technologies Inc. (Riverside, CA, USA). Because these are proprietary products, we can not disclose the specific components or their proportions in these formulations that we utilized for various experiments.

### Two-choice bioassay: Experiment setup

The two-choice trap bioassay setup followed in our study was based on the methods described by Babu et al. [17]. Briefly, each bioassay chamber was prepared using a (1839 mL) clear polyethylene container (Dart Container Corporation, Mason, MI) with dimensions of 19.7 × 20.6 × 7.8 cm, fitted with a matching clear lid. The lid had two circular openings, 2 cm in diameter each. One opening was secured with a mesh covering for air circulation. The other was sealed with a cotton ball, serving as an insect release point before the assay. Two *D*. *suzukii* traps, each made with 59-mL clear plastic portion cups (Fabri-Kal Corp., Kalamazoo, MI) with a fitting lid, were glued diagonally inside the chamber bottom. A single 1.6-cm-diameter hole was drilled at the center of the trap lid. This opening was fitted with an inverted rubber cap from a floral water pick (DL 3805, Diamond line containers, Akron, OH). This water pick cap had a single 3-mm-diameter opening in the center to serve as the *D*. *suzukii* entry point to the traps. The cap of the water pick was inserted into the trap lid such that the upper surface of the cap projected into the trap while the bottom edge of the rubber cap was in line with the upper surface of the trap lid. Three circular cotton pads (Swisspers, Gastonia, NC) were arranged between the traps. Before the chamber was closed, the cotton pads were moistened with 8 mL of distilled water to increase the humidity inside the bioassay arena. Additionally, a floral tube (Juvo Plus Inc., Monrovia, CA) filled with distilled water and secured with a moisturized cotton ball, placed in the bottom center of the arena, served as the additional water source for the flies during the assay. All two-choice bioassays were performed only at the University of Georgia, Athens, GA.

### Two-choice bioassay: Comparing *D*. *suzukii* attraction to TD and OR1 formulations

To confirm the ability of TD and OR1 formulations to attract adult *D*. *suzukii* and to quantify the relative attraction of flies between these formulations, we conducted a series of two-choice assays. We evaluated the ability of the OR1 and TD formulations to attract adult *D*. *suzukii*. We tested insect attraction to the TD or OR1 formulation against a distilled water control. Additionally, for a direct comparison of *D*. *suzukii* attraction among TD and OR1 formulations, adult *D*. *suzukii* relative attractiveness to the above formulations was tested against each other. Because both TD and OR1 are formulated as adjuvants without any insecticide component, we further compared the *D*. *suzukii* relative attractiveness to the A&K formulations prepared using these adjuvants by mixing the adjuvants with 0.25% spinosad. All the treatment pairs were replicated six times in a randomized complete block design. The laboratory benches, that differ in the natural light availability due to their relative position to the glass windows on one side of the room, considered as blocking factor.

### Two-choice bioassay: Presence of dye and addition of spinosad on the attraction of TD and OR1 formulations to adult *D*. *suzukii*

The ACTTRA SWD experimental formulations contain a red dye for adult *D*. *suzukii* visual attraction. Additionally, as adjuvants, TD and OR1 formulations needed to be mixed with a suitable insecticide to prepare the A&K formulation. Therefore, to test the influence of dye and spinosad on the relative attractiveness of the formulations to adult *D*. *suzukii*, we compared the attractiveness of both the TD and OR1 formulations with and without dye, and with and without spinosad (0.25% spinosad (v/v) Entrust™ 22.5 SC) against blueberry fruit as an alternate choice. Additionally, we included a water control and HOOK SWD as treatments to test against the blueberry fruit choice. All the treatment pairs were replicated six times and arranged as randomized complete block designs.

For all the two-choice assays involved with ACTTRA SWD formulations, an application of 80 μL of 87.5% treatment served as the choice. Prior studies identified this volume as the optimum ACTTRA SWD treatment volume for similar two-choice assays performed with the same arena [17]. Similarly, 80 μL of distilled water served as the water control. When blueberry fruit was presented, 15 g of fruits served as the choice. To avoid any positional biases among the two choices in an arena across replications, treatment choices were randomly placed in one of the two choice traps in the bioassay chamber. Twenty adult *D*. *suzukii* (5–7 days old; 1: 1 sex ratio), starved for ~2 h, were released into each arena. The number of *D*. *suzukii* in each of the traps that responded to each treatment was counted 24 h after the insect release. Bioassays were conducted in a laboratory maintained at 21 ± 2°C and 24 h light. Relative humidity inside the two-choice arena was recorded as 90 ± 2%.

### No-choice bioassays: Experiment setup

The no-choice bioassays were replicated at three laboratories located in the following three US states: Georgia (GA), New Jersey (NJ), and Florida (FL). The bioassay arena was prepared with a (946 mL) clear plastic container (Fabri-Kal^®^, Kalamazoo, MI) with a matching lid. Each arena contained a single 5- to 7-cm-long blueberry terminal with 3–4 leaves inserted into a water pick with distilled water (DL 3805, Diamond Line, Akron, OH) (GA and NJ), or terminals were surrounded by a foam stopper and inserted into the bottom half of a 50-mL centrifuge tube with distilled water (FL). Water picks were fitted through a circular hole in the bottom center of the arena while the centrifuge tubes were placed inside the arena. As a food source and oviposition substrate, the assay container was supplied with 13 ripe organic blueberries (Simple Truth Organic, Kroger Co. Cincinnati, OH). In addition, a floral tube (GA and NJ) (Juvale, Juvo Plus Inc. Monrovia, CA) or plastic test tube (FL) (Fisher Scientific International, Inc. Hampton, NH) filled with distilled water and with its opening plugged with moist cotton was provided as a water source.

In the GA study, the blueberry terminals were collected from a grower’s field near Alma, GA, USA (31.5446, -82.3893), planted with the Southern highbush blueberry variety ‘Meadowlark.’ Since the grower applied Sulforix (Tessenderlo Kerley Inc., Phoenix, AZ), at a rate of 14 L/ha, 4 days prior to the experiment, the plant terminals were washed thoroughly to remove any potential residue. For this step, terminals were first immersed in mild unscented detergent solution for 15 minutes, followed by rinsing in tap water for three times and a final rinse in distilled water. For the FL study, the blueberry terminals were collected from a research plot at the Plant Science Research and Education Unit located at Citra, FL, planted with the Southern highbush blueberry variety ‘Farthing.’ Similarly, for the NJ study, the terminals were collected from a research plot at the Rutgers P.E. Marucci Center located in Chatsworth, NJ, planted with the Northern highbush blueberry variety ‘Bluecrop.’ The research plots in FL and NJ received no insecticide applications.

The treatments used in this study are: TD and OR1 formulations with and without dye, and with and without spinosad (0.25% spinosad (v/v) Entrust™ 22.5 SC). Additionally, we included an untreated control (UTC), spinosad spray treatment at the field recommended rate (0.438 L/ha of Entrust™ 22.5 SC in 374.2 L/ha of water), and HOOK SWD as treatments. The blueberry terminal in each container, except for that assigned for untreated control or for a direct spinosad spray treatment, received a single 0.2-mL drop of the corresponding ACTTRA SWD formulation, applied to the adaxial surface of the topmost leaf. For the spinosad spray treatment, ~0.52 mL of insecticide solution was applied per blueberry terminal by spraying with a fingertip sprayer (Equate fingertip sprayer 2 fl. oz. [59.15 mL], Wal-Mart Stores Inc., Bentonville, AR). Four sprays per terminal, each delivering around 0.13 mL (± 0.002) of insecticide solution, were applied from different directions around the plant terminal to uniformly cover the entire surface.

### No-choice bioassays: Experiment evaluation

The bioassay containers with treatments were arranged in a randomized complete block design, and each treatment was replicated five (FL and NJ) or six times (GA). Except for FL, five male and five female adult *D*. *suzukii*, 5–7 days old and starved for ~2 h, were released into the bioassay containers. After flies were added, the containers were kept for 6 days in a laboratory maintained at ~21°C,14:10 L:D, and 80 ± 10% RH. On day 6, blueberries were removed from the bioassay containers, transferred to a ventilated (237 mL) deli container (Fabri-Kal^®^, Kalamazoo, MI) over a cotton pad, and incubated under the same lab conditions for 14 days to assess adult progeny emergence. To determine treatment efficacy, we measured adult male and female mortality on days 1, 3, and 6 (days after treatment [DAT]) and the number of adult *D*. *suzukii* progenies that emerged from the blueberry fruits.

### Data analyses

Statistical analyses were performed using GraphPad Software (San Diego, CA), R software version 4.3.1 [28], and SAS v. 9.4 (SAS Institute 2011). Data from all behavioral (two-choice) assays were assessed for the normality of residuals using Shapiro-Wilk test (*α* = 0.05). Data from two-choice assays that passed the normality test were subjected to Student’s two-tailed paired *t*-tests (GraphPad Prism V.9) at *α* = 0.05. The data that violated the normality assumption was analyzed using the Wilcoxon matched-pairs signed rank test (GraphPad Prism V.9, *α* = 0.05). Additionally, the influence of dye and the addition of spinosad on the TD and OR1 formulations’ attraction of adult *D*. *suzukii* in the presence of blueberry host fruits was analyzed using a generalized linear mixed model (PROC GLIMMIX). Means were separated post-analysis of variance (ANOVA) using the Tukey-Kramer test (*α* = 0.05). Additionally, a comparison of means between specific groups of treatments (i.e., ACTTRA SWD formulations with dye *vs*. no dye, and ACTTRA SWD formulations with spinosad *vs*. no spinosad) was carried out using the ESTIMATE statement. Finally, the relative difference in *D*. *suzukii* catch between ACTTRA SWD formulations, HOOK SWD, or water against the blueberry choice was compared using an “Attractivity Index” (AI). AI was calculated as follows: 100 × [(number of flies in the ACTTRA SWD formulations, HOOK SWD, or water treatment − number of flies in the blueberry choice)/(total number of flies trapped)] [29]. A significant departure of AI value from zero was tested using a non-parametric sign test (PROC UNIVARIATE) at *α* = 0.05. A significant positive mean AI value indicates an overall adult *D*. *suzukii* attraction to ACTTRA SWD, HOOK SWD, or water treatment over the blueberries. Conversely, a significant negative mean AI value indicates an overall attraction of adult *D*. *suzukii* to blueberry fruits over the corresponding alternate treatment choice.

For the no-choice (efficacy) bioassay studies, Cox proportional hazards regression was used to assess the association of the state (GA, NJ, FL) and treatments to *D*. *suzukii* adult mortality hazard [30]. The coxph function in the survival package was used to fit the model and to interpret the results [31]. Flies alive at the end of the 6-day observation period were censored at 6 days. Non-significant covariates were removed from the model and refitted. We assessed the linearity and proportional hazards assumptions and checked for separation, collinearity, outliers, and influential observations. No issues were found except that the state predictor had a meaningfully large deviation from the proportional hazards assumption, and we relaxed the assumption by using stratification where each level of the variable (state) is assumed to have a different baseline hazard function but the same coefficients for the other predictors in the model [32].

During the no-choice bioassay, female flies oviposited in blueberries provided within their chamber. The number of adult progenies eclosed from the infested blueberries within each assay chamber was counted. A generalized linear model was used to determine how the number of progeny was predicted by the treatments tested and from the three states. The progeny counts were adjusted for the number of female flies placed inside each chamber. The negative binomial distribution was determined to be appropriate based on the presence of overdispersion. Other model assumptions (e.g., homoscedasticity, linearity, independence) were assessed, and no issues were found. Following the estimation of the above models, pairwise multiple comparison tests were performed using the emmeans function within the emmeans package in R [33]. Additional contrasts were tested to compare *D*. *suzuki*i adult mortality and progeny emergence between TD *vs*. OR1, formulations with dye *vs*. no dye, and with added spinosad *vs*. no spinosad, etc.

## Results

### Two-choice bioassays: Comparing *D*. *suzukii* attraction to TD and OR1 formulations

**Fig 1:**
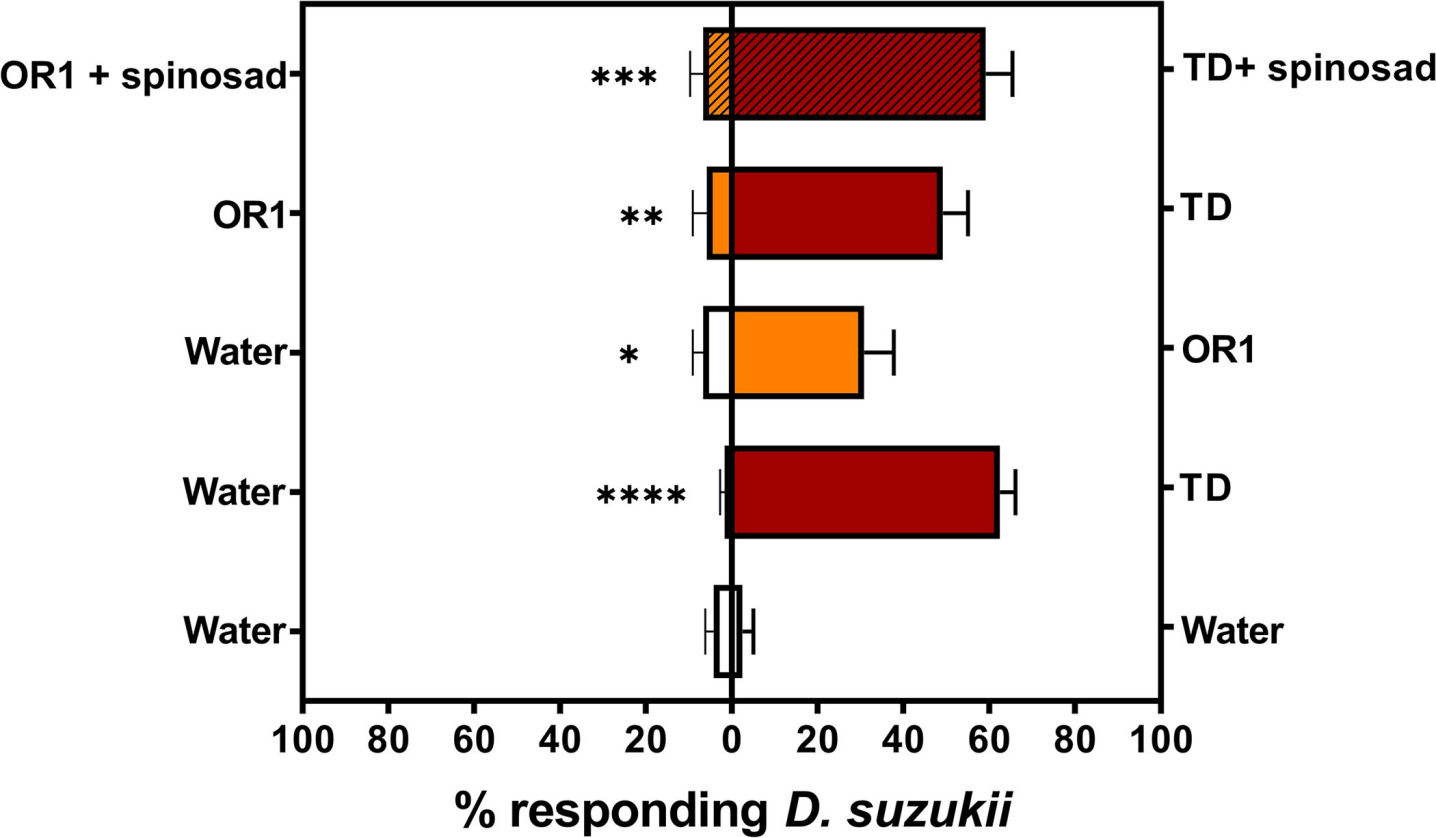
Mean (± SEM) percent of total *D*. *suzukii* released in an arena captured inside the traps that contained water control vs. water control, TD or OR1 *vs*. water control, TD vs. OR1, and TD + spinosad *vs*. OR1 + spinosad. Significant statistical difference is shown (paired *t*-test; *α* = 0.05); * (*P*<0.1), ** (*P*<0.01), *** (*P*<0.001), and **** (*P*<0.0001).

The mean percentage of total adult *D*. *suzukii* released in an arena that responded to the traps containing distilled water was minimal, ranging from 1.67 ± 1.05 to 6.67 ± 2.47 (% mean ± SEM). No significant difference in adult *D*. *suzukii* response was observed when both choices were distilled water (*t* = 0.79; df = 5; *P* = 0.4650; Fig 1). However, compared with the water control, both TD and OR1 formulations were significantly more attractive to adult *D*. *suzukii* (TD, *t* = 13.95; df = 5; *P* < 0.0001; OR1, *t* = 3.868; df = 5; *P* = 0.0118). A direct comparison between TD and OR1 formulations indicated that the TD formulation was ~8 times more attractive than OR1 (*t* = 6.50; df = 5; *P* = 0.0013). Similarly, the TD formulation + spinosad was ~9 times more attractive than the OR1 formulation + spinosad (*t* = 8.72; df = 6; *P* = 0.0003).

### Two-choice bioassays: Presence of dye and addition of spinosad on TD and OR1 formulations’ attraction to adult *D*. *suzukii*

**Fig 2:**
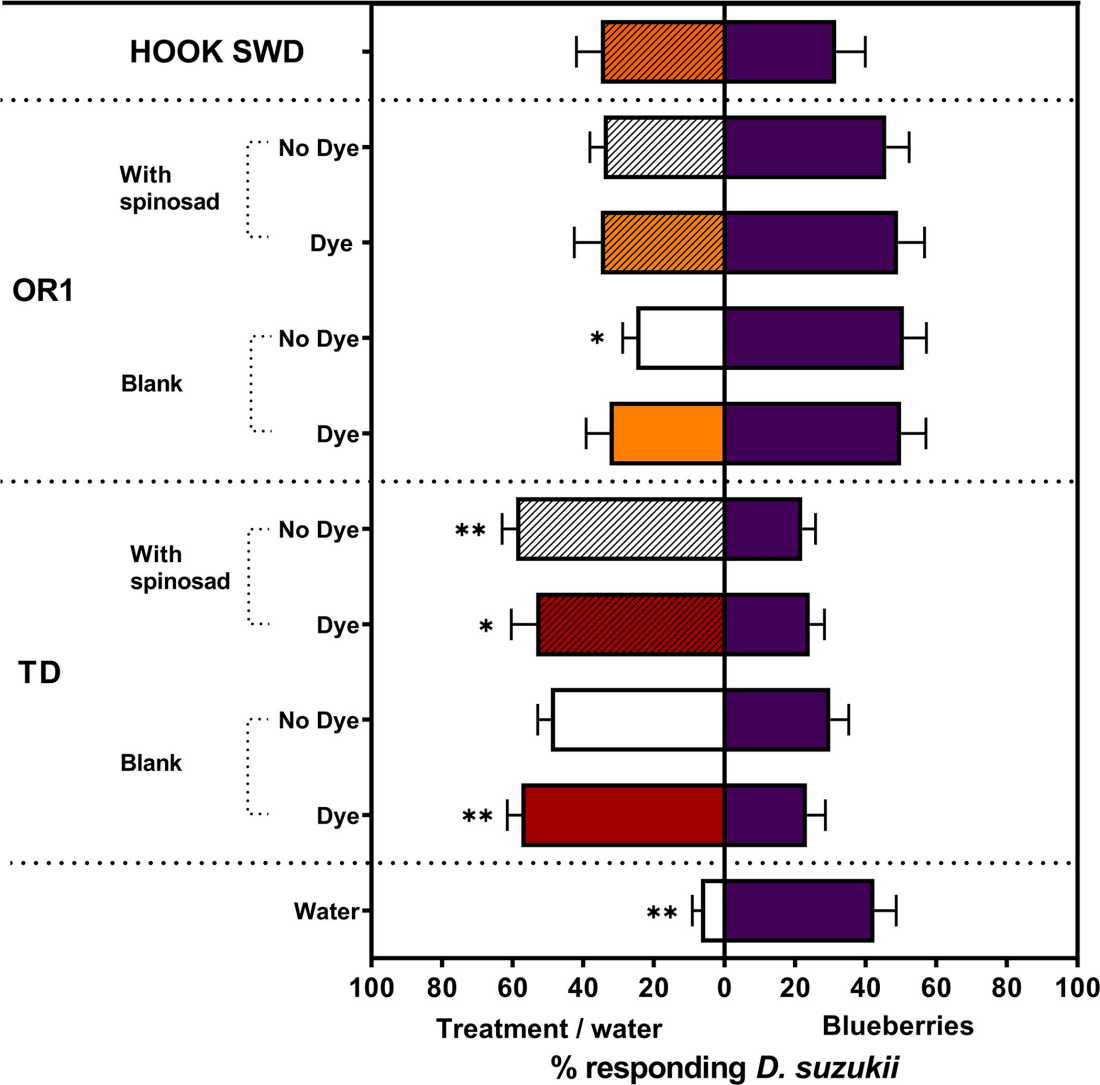
Mean (± SEM) percentage of total *D*. *suzukii* released in an arena captured inside the traps that contained various ACTTRA SWD treatments vs. blueberry fruits. Significant statistical difference is shown (paired *t*-test or Wilcoxon matched-pairs signed rank test; *α* = 0.05); * (*P*<0.05), and ** (*P*<0.01).

For adult *D*. *suzukii*, blueberry fruits were significantly more attractive than the water control (*t* = 4.95; df = 5; *P* = 0.0043; Fig 2). When blueberries were provided as an alternate choice, adult *D*. *suzukii* showed significantly greater attraction to the TD formulations except for TD with no dye (TD (no dye), *t* = 2.25; df = 5; *P* = 0.0740; TD (dye), *t* = 4.22; df = 5; *P* = 0.0084; TD (no dye) + spinosad, *t* = 5.74; df = 4; *P* = 0.0046; and TD (dye) + spinosad, *W* = −21; *P* = 0.0313; Fig 2). In contrast, none of the OR1 formulations tested was more attractive than the corresponding blueberry choice (OR1 (dye), *t* = 1.38; df = 5; *P* = 0.2266; OR1 (no dye) + spinosad, *t* = 1.18; df = 5; *P* = 0.2911; and OR1 (dye) + spinosad, *t* = 0.99; df = 5; *P* = 0.3686; Fig 2). Moreover, the OR1 (no dye) formulation was significantly less attractive than the corresponding blueberry choice (*t* = 2.91; df = 5; *P* = 0.0336). Similarly, no significant difference in *D*. *suzukii* attraction was observed between HOOK SWD and the blueberry fruit choice (*t* = 0.24; df = 5; *P* = 0.8198).

**Fig 3:**
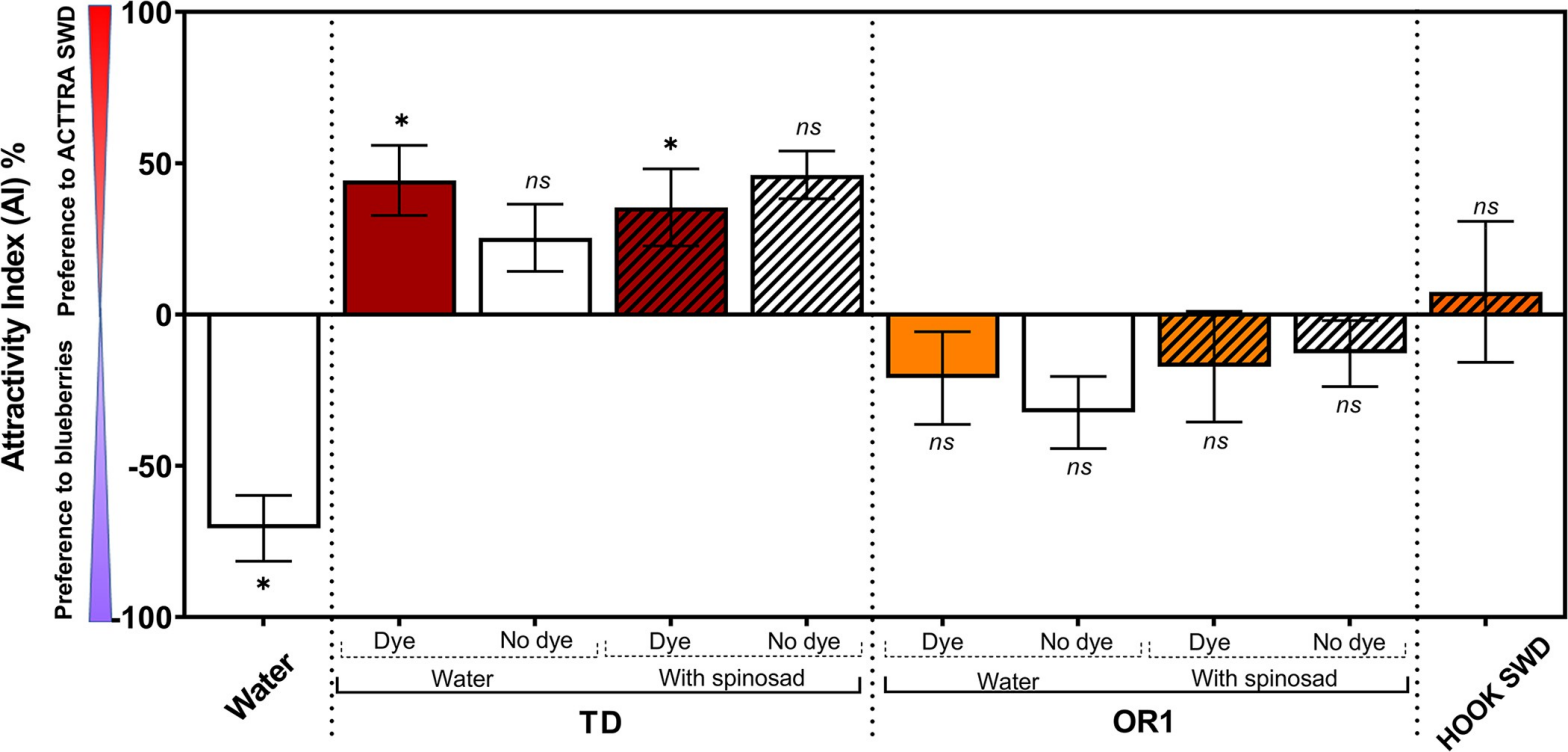
The attractivity index (AI) in percentage. Significant statistical difference from zero is shown (sign test; *α* = 0.05); *ns* (*P*>0.05), and * (*P*<0.05). A significant positive mean AI value indicates an overall adult *D*. *suzukii* attraction to TD, OR1, HOOK SWD, or water, depending on treatment over the alternate choice of blueberries. A significant negative mean AI value indicates an overall attraction of adult *D*. *suzukii* to blueberry over the corresponding treatment choices.

All the treatments with the TD formulation showed net positive AI values. However, only for TD (dye) and TD (dye) + spinosad, the mean positive AI values were significantly different from ‘0,’ indicating a significant mean attraction of adult *D*. *suzukii* to the TD treatments over the ripe blueberry fruits (TD (no dye), *M* = 2.00; *P* = 0.2188; TD (dye), *M* = 3.00; *P* = 0.0313; TD (no dye) + spinosad, *M* = 2.50; *P* = 0.0625; and TD (dye) + spinosad, *M* = 3.00; *P* = 0.0313; Fig 3). All the treatments with OR1 formulation showed net negative AI values. However, none of the mean AI values were significantly different from ‘0’ (OR1 (no dye), *M* = −2.00; *P* = 0.2188; OR1 (dye), *M* = 0.00; *P* = 1.00; OR1 (no dye) + spinosad, *M* = −1.00; *P* = 0.6875; and OR1 (dye) + spinosad, *M* = −1.50; *P* = 0.3750). HOOK SWD showed a net positive AI value, although the mean was not significantly different from ‘0’ (*M* = −0.50; *P* = 1.0000). Finally, for adult *D*. *suzukii*, blueberry fruits were significantly more attractive than the water control (*M* = −3.00; *P* = 0.0313).

Based on AI values, a comparison of the response of adult *D*. *suzukii* to TD and OR1 formulations in the presence of a blueberry fruit choice indicates that, overall, the treatments with TD formulation were significantly more attractive to adult *D*. *suzukii* than the treatments containing OR1 formulation (TD *vs*. OR1, *t* = 5.81; df = 43.98; *P* < 0.0001; Fig 3). Irrespective of the ACTTRA SWD formulation and the presence or absence of spinosad, the addition of dye to the treatments did not significantly alter the attraction of adult *D*. *suzukii* compared with the treatments that lacked the dye (dye *vs*. no dye, *t* = 0.37; df = 43.98; *P* = 0.7095). Similarly, irrespective of dye status, overall, the addition of spinosad to either TD or OR1 formulations did not influence the adult *D*. *suzukii* response to these formulations (TD + spinosad *vs*. TD, *t* = 0.41; df = 44.19; *P* = 0.6831; OR1 + spinosad *vs*. OR1, *t* = 0.82; df = 43.76; *P* = 0.4142).

### No-choice bioassays

**Fig 4:**
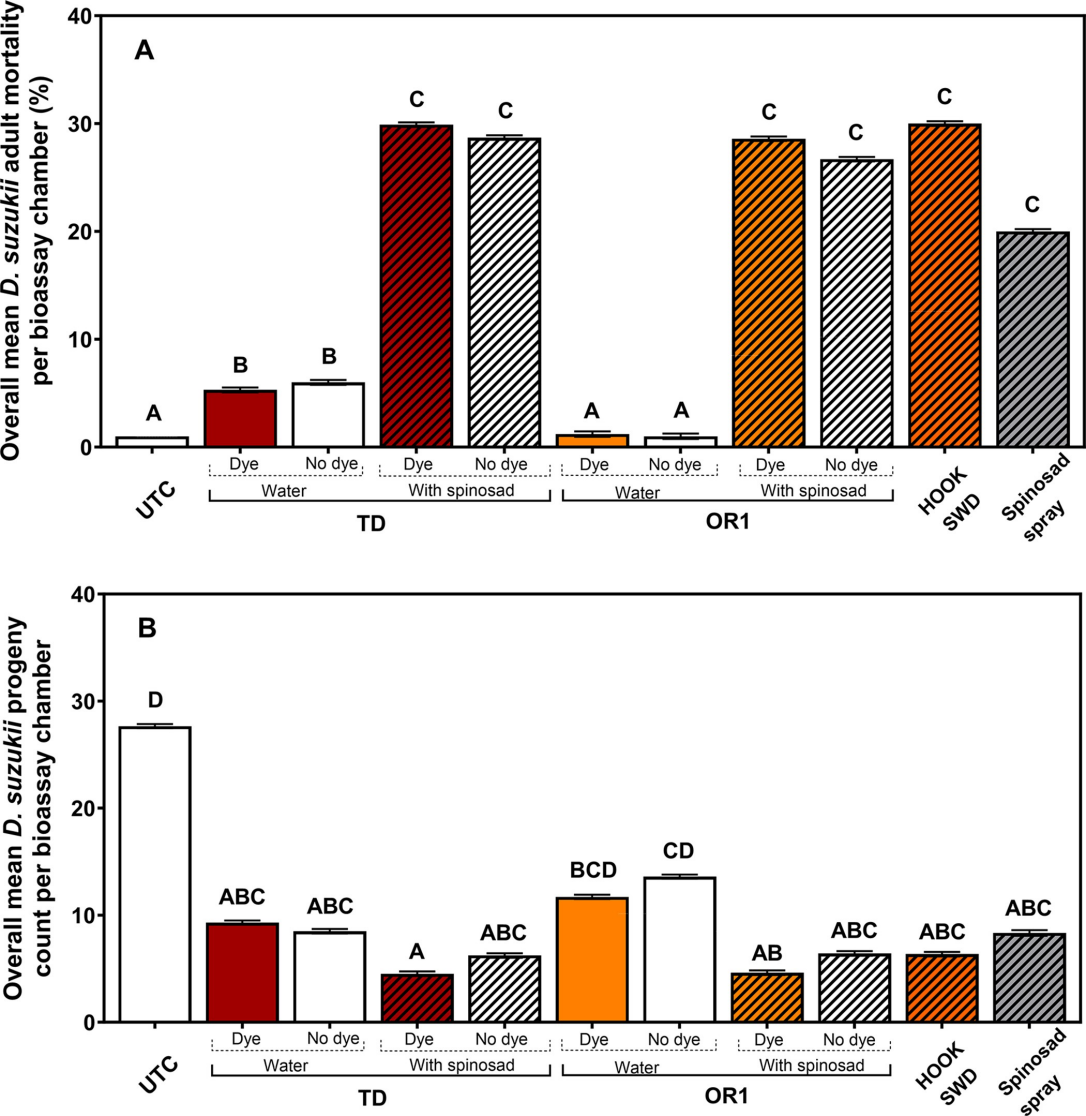
Overall percent mean mortality (± SEM) of adult *D*. *suzukii* (A), and the number of progeny (± SEM) (B) emerged from blueberries per arena in laboratory no-choice bioassay trial with different treatments. The experiment was replicated in GA, NJ, and FL laboratories.

Across the 3 states and the observation dates, the overall percent mean mortality of adult *D*. *suzukii* was significantly influenced by the treatments (*χ*^*2*^ = 822.9; df = 10; *P* < 0.0001; Fig 4A). However, the effect of *D*. *suzukii* sex on mortality was not significant and was removed from the analysis (*χ*^*2*^ = 3.21; df = 1; *P* = 0.07326). Compared with the untreated control, a significantly high *D*. *suzukii* mortality was observed for most treatments, except for OR1 (no dye) and OR1 (dye) treatments (Fig 4A). Overall, the treatments with the TD formulation resulted in significantly higher mortality than the OR1 formulation (TD *vs*. OR1, *Z* = 9.43; df = Inf; *P* < 0.0001). Interestingly, TD formulations irrespective of dye status (TD (dye) + TD (no dye)), even when not added with spinosad, on average, resulted in significantly higher mortality than the corresponding OR1 formulations (*Z* = 10.26; df = Inf; *P* < 0.0001). Within the TD and OR1 treatment groups, the addition of spinosad to the adjuvants resulted in significantly higher *D*. *suzukii* mortality than the treatments with a blank adjuvant (TD + spinosad *vs*. TD, *Z* = 15.97; df = Inf; *P* < 0.0001; OR1 + spinosad *vs*. OR1, *Z* = 21.13; df = Inf; *P* < 0.0001). Overall, irrespective of the specific ACTTRA SWD formulations, the presence of a dye in the formulations significantly enhanced *D*. *suzukii* mortality (dye *vs*. no dye, *Z* = 3.63; df = Inf; *P* = 0.0003). However, within the TD and OR1 treatment groups, irrespective of spinosad presence, the addition of dye to the adjuvants did not significantly influence the *D*. *suzukii* mortality (TD + dye *vs*. TD, *Z* = -0.413; df = Inf; *P* = 0.6794; OR1 + dye *vs*. OR1, *Z* = 0.849; df = Inf; *P* = 0.3961). The regression result from the Cox proportional hazards model is shown in the S1 Table.

A significant treatment effect was observed in the adult *D*. *suzukii* progeny counts that emerged from blueberries exposed to various treatments (*F* = 6.81; df = 10, 156; *P* < 0.0001; Fig 4B). Additionally, the effect of state was also significant (*F* = 145.87; df = 2, 156; *P* < 0.0001). Similar to the adult mortality data, except for the OR1 (no dye) and OR1 (dye), all treatments, including TD (dye) and TD (no dye), resulted in a significant reduction in the *D*. *suzukii* progeny emergence compared with the untreated control (Fig 4B). While TD formulations (TD (dye) + TD (no dye)), even when not added with spinosad, on average, resulted in significantly higher adult *D*. *suzukii* mortality than the corresponding OR1 formulations, such difference was not observed in progeny counts from these treatments (*Z* = -1.71; df = Inf; *P* = 0.0881). Irrespective of the type of formulation or presence of insecticide, the presence of dye in the treatment did not influence the overall adult emergence from the blueberries (dye *vs*. no dye, *Z* = −1.38; df = Inf; *P* = 0.1686). Moreover, the type of formulation, TD or OR1, did not significantly influence progeny emergence (TD *vs*. OR1, *Z* = -1.28; df = Inf; *P* = 0.2017). However, the addition of spinosad to the TD or OR1 formulations significantly reduced *D*. *suzukii* adult progeny emergence (TD + spinosad *vs*. TD, *Z* = -2.45; df = Inf; *P* = 0.0143; OR1 + spinosad *vs*. OR1, *Z* = -4.05; df = Inf; *P* = 0.0001).

## Discussion

Identifying a superior A&K tactic will likely increase the adoption of behavior-based strategies for managing the invasive *D*. *suzukii* and reduce insecticide use in small fruit crops. HOOK SWD has been the most tested SPLAT-based A&K formulation against *D*. *suzukii* [13–16]. HOOK SWD applications have shown promise in reducing *D*. *suzukii* populations in blueberry and raspberry fields when deployed as a part of an IPM program [14, 16]. Our study compared the attractiveness of two SPLAT-based ACTTRA SWD formulations—OR1, which has the same attractant blend as in HOOK SWD, and TD, which contains a newly developed attractive blend. Of the three SPLAT-based A&K products, our study shows that the TD formulation is superior in attracting adult *D*. *suzukii*. A direct two-choice comparison of the attractiveness of the TD formulation with the OR1 formulation shows that the TD formulation is ~8 times more attractive than OR1. Moreover, comparing the ACTTRA SWD formulations mixed with the insecticide spinosad revealed similar results, with TD + spinosad being ~9 times more attractive to adult *D*. *suzukii* than OR1 + spinosad. This improvement in formulation attractivity also translated into *D*. *suzukii* adult mortality in our laboratory no-choice (efficacy) assays. Overall, the TD formulations resulted in higher, or at least a comparable, mortality to the OR1 formulation. Additionally, our results are in agreement with a previous related study [17], which suggested that the TD formulation is better than the OR1 formulation at attracting adult *D*. *suzukii* when host fruits are present. Overall, our laboratory results demonstrated a higher efficacy of a TD-based A&K product in managing *D*. *suzukii* over its well-tested predecessor, the OR1 formulation.

It has been reported that the attraction of adult *D*. *suzukii* to SPLAT-based A&K formulations is highly context-dependent [16, 17]. Our two-choice assays comparing adult *D*. *suzukii* attraction to the OR1 and TD formulations indicate the higher attractivity of the TD formulation over the OR1 formulation in a laboratory setting. However, in the field, the attraction of adult *D*. *suzukii* to these A&K formulations can be influenced by the presence of host fruit volatiles as well as by the sex and physiological state (i.e., age and mating status) of the flies [17]. Host fruit volatiles will likely to compete with the A&K formulations for *D*. *suzukii* attraction under field conditions [16]. Thus, in our study, we compared the attractiveness of the OR1 and TD formulations to adult *D*. *suzukii* when blueberry fruit was present as an alternate choice. Notably, the complete A&K formulation prepared with TD (i.e., TD + dye + spinosad) showed a competitive edge over the ripe blueberries for *D*. *suzukii* attraction, as adult flies preferred this A&K mixture over the fruits. This finding indicates the potential of the TD-based A&K formulation for managing *D*. *suzukii* population when blueberry fruits are present in the field. In contrast, *D*. *suzukii* flies showed no clear preference for HOOK SWD or the A&K mixture prepared with the OR1 formulation (i.e., OR1 + dye + spinosad) over the fruits. These data support a previous study showing that the ability of HOOK SWD to suppress *D*. *suzukii* populations breaks down with increasing host fruit densities [16].

Interestingly, our no-choice efficacy bioassay results indicate that the blank TD formulations (without added insecticide but with or without added dye) resulted in higher adult *D*. *suzukii* mortality than the blank OR1 formulations. This suggests that the TD formulation, without any added insecticide, is inherently more lethal to adult *D*. *suzukii* than the OR1 formulation. However, the higher *D*. *suzukii* adult mortality observed in the blank TD formulations over blank OR1 formulations did not translate into a significantly lower progeny emergence in blank TD than blank OR1 formulations. Thus, the slight inherent lethality of TD formulation is likely not sufficient alone in resulting acceptable levels of *D*. *suzukii* control. The addition of an insecticide, like spinosad, is, therefore, necessary for attaining adequate *D*. *suzukii* control when the product is deployed in the field as a *D*. *suzukii* management strategy.

Both A&K formulations, OR1 and TD, retained their attractiveness to adult *D*. *suzukii* after they were mixed with the insecticide spinosad as the killing agent. Adding an insecticide to an attractive blend when preparing A&K mixtures can result in an overall change in the product’s attractivity to the target insect [5]. For example, the addition of zeta-cypermethrin (Mustang Maxx) to OR1 improved the attractiveness of the resulting formulation to adult *D*. *suzukii*, while the addition of azadirachtin + pyrethrins (Azera) to both OR1 and TD reduced the A&K formulations’ attractiveness [34]. Notably, pyrethrum extract has been proven to repel adult *Drosophila melanogaster* Meigen, a species taxonomically closely related to *D*. *suzukii* [35]. In our experiments, the addition of spinosad, the default insecticide component in the SPLAT-based A&K formulations such as HOOK SWD, with OR1 and TD did not significantly change the formulations’ attractiveness to adult *D*. *suzuki*i. Moreover, our results are consistent with the previous research, where the addition of spinosad to either OR1 or TD did not result in a significant change in the adult *D*. *suzukii* behavioral response to these A&K formulations [34].

Adult *D*. *suzukii* color perception studies indicate that the flies are predominantly sensitive to the light spectra in the UV range and then to orange-red wavelengths [36]. Accordingly, results from several studies on trap color optimization also support the above preference where adult flies are attracted to monitoring traps with red and black colors and also to red and black strips, yellow, and purple colors [21–24]. These color preferences match their host fruit colors, namely, red for strawberries, raspberries, and cherries; purple for blueberries; and black for blackberries [24]. Accordingly, by default, the ACTTRA SWD formulations contain a red dye (often described as pink in the literature [14]) for insect visual attraction. However, our laboratory choice (behavior) and no-choice (efficacy) studies indicate that adding a red dye to the A&K formulations did not increase the formulations’ attractiveness to adult *D*. *suzukii* or reduce progeny emergence. While overall, the addition of a dye to SPLAT formulations led to higher adult *D*. *suzukii* mortality compared with the no-dye formulations, this effect was not observed when comparisons were made within the TD or OR1 formulations. In fact, recent *D*. *suzukii* color perception studies suggest that adult flies have a limited capability to distinguish red color, and the color contrast between the foreground and background colors may be more critical in color perception [20, 25]. Therefore, in our study, the treatment with the red A&K formulation inside a transparent arena arranged over a black laboratory bench likely provided little contrast between foreground (red) and background (black) colors for adult *D*. *suzukii* color perception [24, 25]. This poor color contrast between foreground and background colors might be the reason for no improvement in the *D*. *suzukii*’s behavioral response for the A&K formulations with red dye over the corresponding blank formulation, a white blank formulation arranged on a black background, in our laboratory assays. We expect that a better color contrast between the red ACTTRA A&K formulation with the background could be achieved under field conditions.

In summary, the results from our study indicate that, compared with the previous attractive blend in the OR1 formulation, the newer blend in the TD formulation is better for attracting adult *D*. *suzukii*, which resulted in overall higher fly mortality. Furthermore, our results, along with previous related studies, indicated that the TD formulation exhibited higher competitiveness for *D*. *suzukii* attraction in the presence of host fruit. Thus, between the OR1 and TD formulations, this study has identified the TD formulation as a better candidate for the development of A&K strategies for managing *D*. *suzukii*. These findings will likely accelerate the development, implementation, and grower adoption of behavior-based tactics, such as A&K, as an IPM component for the management of *D*. *suzukii* in small fruit crops.

## Supporting information

S1 Table Regression results from the Cox proportional hazards model. (DOCX)

S1 Data Data underlying the figures in this study. (XLSX)
