# Supplementary material for: Comparison of multimodal attract-and-kill formulations for managing Drosophila suzukii: Behavioral and lethal effects
Source: PLoS One. 2023 Dec 7;18(12):e0293587. doi: 10.1371/journal.pone.0293587 (PMC10703201; doi:10.1371/journal.pone.0293587)
Supplement: S1 Table — (DOCX) [file pone.0293587.s001.docx]

**Supporting information (Table S1)**

Table S1: Regression results from Cox proportional hazards model

| Cox regression results for time to adult *D. suzukii* death for a treatment (N = 1613) | | | | | |
| --- | --- | --- | --- | --- | --- |
| Factor | Regression Coefficients | | Adjusted Hazard Ratio | | p-value |
|  | Log(HR)^1^ | 95% CI^1^ | HR^1^ | 95% CI^1^ |  |
| Treatment |  |  |  |  |  |
| Untreated Control | - | - | - | - |  |
| TD (dye) | 1.673 | 1.237, 2.109 | 5.326 | 3.444, 8.237 | <0.001 |
| TD (no dye) | 1.788 | 1.346, 2.229 | 5.976 | 3.843, 9.293 | <0.001 |
| OR1 (dye) | 0.188 | -0.303, 0.679 | 1.207 | 0.738, 1.972 | 0.453 |
| OR1 (no dye) | 0.035 | -0.457, 0.526 | 1.035 | 0.633, 1.692 | 0.890 |
| TD (dye) + Spinosad | 3.400 | 2.988, 3.811 | 29.950 | 19.838, 45.215 | <0.001 |
| TD (no dye) + Spinosad | 3.359 | 2.945, 3.772 | 28.749 | 19.009, 43.481 | <0.001 |
| OR1 (dye) + Spinosad | 3.355 | 2.940, 3.769 | 28.640 | 18.921, 43.351 | <0.001 |
| OR1 (no dye) + Spinosad | 3.284 | 2.869, 3.699 | 26.677 | 17.614, 40.403 | <0.001 |
| Spinosad | 2.994 | 2.576, 3.413 | 19.971 | 13.145, 30.341 | <0.001 |
| Hook SWD | 3.402 | 2.987, 3.816 | 30.011 | 19.834, 45.410 | <0.001 |
| ^1^ HR = Hazard Ratio, CI = Confidence Interval | | | | | |
